# Supplementary material for: Identification and functional analysis of non-coding regulatory small RNA FenSr3 in Bacillus amyloliquefaciens LPB-18
Source: PeerJ. 2023 May 15;11:e15236. doi: 10.7717/peerj.15236 (PMC10194069; doi:10.7717/peerj.15236)
Supplement: Supplemental Information 4 [file peerj-11-15236-s004.zip › KO/CK-vs-T1_map/map04214.html]

KEGG PATHWAY: Apoptosis - fly - Reference pathway


|  |  |
| --- | --- |
| **Apoptosis - fly - Reference pathway** |  |

[
Pathway menu
| Organism menu
| Pathway entry
| Show description
| User data mapping
]

Apoptosis, or programmed cell death, is a critical factor in homeostasis in all multicellular organisms, playing a crucial role in removing excess unwanted cells without stimulation of an immune response. Caspases are the central components of the apoptotic response. In Drosophila, Dark promotes activation of the initiator caspase Dronc. Drosophila inhibitor of apoptosis protein 1 (DIAP1) inhibits Dronc and the effector caspases Drice and Dcp-1. RHG family proteins such as Rpr, Hid, Grim, Sickle, and Jafrac-2 promote cell death, in part by disrupting DIAP1's ability to inhibit caspase activity. Proapoptotic proteins such as caspases and RHG proteins are regulated through multiple pathways. The Drosophila steroid hormone ecdysone mediates cell death by controling the up-regulation of a number of proapoptotic genes. Cytotoxic stress such as DNA damage can activate P53, which in turn induces the expression of RHG genes. Eiger can initiate cell death through an IAP-sensitive cell death pathway via JNK signaling. Hid, a member of the RHG family, is negatively regulated by the epidermal growth factor (EGF) receptor-dependent survival signaling and positively regulated by the tumor suppressors Hippo, Salvador, and Warts.

|  |  |  |
| --- | --- | --- |
| Reference pathway | 184% 150% 122% 100% 82% 67% 55% | 图片下载 |
